# IgM型多发性骨髓瘤六例报告并文献复习

**DOI:** 10.3760/cma.j.issn.0253-2727.2021.12.008

**Published:** 2021-12

**Authors:** 文强 严, 慧守 樊, 婧钰 许, 佳慧 刘, 辰星 杜, 书会 邓, 伟薇 隋, 燕 徐, 录贵 邱, 刚 安

**Affiliations:** 中国医学科学院血液病医院（中国医学科学院血液学研究所），实验血液学国家重点实验室，国家血液系统疾病临床医学研究中心，天津 300020 State Key Laboratory of Experimental Hematology, National Clinical Research Center for Blood Diseases, Institute of Hematology & Blood Diseases Hospital, Chinese Academy of Medical Sciences & Peking Union Medical College, Tianjin 300020, China

**Keywords:** 多发性骨髓瘤, 免疫球蛋白M, 高黏滞血症, Multiple myeloma, Immunoglobulin M, Hyperviscosity

## Abstract

**目的:**

探究IgM型多发性骨髓瘤（MM）患者的临床特征、疗效及预后。

**方法:**

收集中国医学科学院血液病医院2009年12月18日至2020年10月29日收治的6例IgM型MM患者临床资料，对其临床特点、实验室检查结果、骨髓检查结果、疗效及预后进行总结、分析。

**结果:**

6例患者均符合IgM型MM的诊断标准。男4例，女2例，初次诊断中位年龄为70（59～81）岁。Durie-Salmon（DS）分期：ⅠA期2例，ⅢA期4例；国际分期系统（ISS）分期：Ⅱ期4例，Ⅲ期2例。初发症状：骨痛4例，高黏滞血症3例，淋巴结肿大或肝脾大2例。实验室检查：中位血清M蛋白39.11（3.61～75.56）g/L，中位血清IgM 69.35（4.35～137.00）g/L，中位HGB 87（70～131）g/L，中位血清肌酐83.6（53.0～129.6）µmol/L，中位血清钙2.12（2.11～2.50）mmol/L。中位骨髓浆细胞比例为0.390（0.255～0.590），4例患者外周血细胞形态学可见浆细胞。染色体核型分析：低二倍体1例。FISH检查：2例P53基因缺失，1例1q21扩增阳性，4例RB-1基因缺失阳性；6例患者IgH重排均阳性，其中3例CCND1/IgH融合基因阳性，明确为t（11;14）重排改变。免疫表型：CD38、CD138、单克隆轻链表达均阳性，4例CD20个别阳性。6例患者均接受蛋白酶体抑制剂为主的方案治疗，达到部分缓解至严格意义的完全缓解，治疗敏感性尚可。

**结论:**

IgM型MM除骨髓瘤典型临床表现外，可伴高黏滞血症、淋巴结肿大或肝脾大等特征性表现，细胞遗传学以t（11;14）多见治疗反应及预后与常见骨髓瘤亚型无明显区别。

## Abstract (en)

**Objective:**

To investigate the clinical characteristics, responses, and prognosis of immunoglobulin M multiple myeloma (IgM MM).

**Methods:**

The clinical characteristics, laboratory results, bone marrow biopsy results, response, and prognosis of six cases of IgM MM in the Blood Diseases Hospital, Chinese Academy of Medical Sciences, from December 18, 2009 to October 29, 2020 were collected and analyzed.

**Results:**

All six cases met the diagnosis criteria of IgM MM. There were four males and two females. The median age at first diagnosis was 70 (59–81) years. According to Durie-Salmon (DS) staging, 2 cases were in IA, and 4 cases were in IIIA. According to the International Staging System (ISS), 4 cases were in II, and 2 cases were in III. The initial symptoms were as follows: 4 cases of bone pain, 3 cases of hyperviscosity, and 2 cases of lymphadenopathy or hepatosplenomegaly. Laboratory results showed the following: median blood M protein: 39.11 (3.61–75.56) g/L; median serum IgM: 69.35 (4.35–137.00) g/L; median hemoglobin: 87.0 (70–131) g/L; median blood creatinine: 83.6 (53.0–129.6) µmol/L; median blood calcium: 2.12 (2.11–2.50) mmol/L. The median ratio of bone marrow plasma cells was 0.390 (0.255–0.590), and in four cases, plasma cells were observed in blood smears. Karyotype analysis and fluorescence in situ hybridization (FISH) examination showed the following: 1 case of hypodiploidy, 2 cases of P53 gene deletion, 1 case of 1q21 amplification positive, and 4 cases of RB-1 gene deletion positive. The immunoglobulin heavy chain (IgH) rearrangement was positive in all cases, of which 3 cases were CCND1/IgH fusion gene-positive identified with t(11;14) rearrangement. Immunophenotyping revealed that all cases were positive for CD38, CD138, and monoclonal light chain and four cases were weakly positive for CD20. All cases accepted proteasome inhibitor-based regimens and attained the response of partial remission to strict complete remission.

**Conclusion:**

In addition to the typical clinical manifestations of myeloma, IgM MM is also characterized by hyperviscosity, lymphadenopathy, or hepatosplenomegaly, and t(11;14) is the most frequent cytogenetics aberration. Furthermore, the response and prognosis of IgM MM are similar to other common myeloma subtypes.

多发性骨髓瘤（multiple myeloma，MM）是一种以浆细胞异常增殖为特征的血液系统恶性肿瘤，分泌大量单克隆免疫球蛋白为其主要特征[1]，其中分泌IgM的MM非常罕见，仅占0.5％～0.8％[2]–[3]，其临床特征及预后不详，国内尚未见大系列报道。本文对我中心6例IgM型MM患者进行回顾性分析及文献复习，以了解IgM型MM患者的临床特征、疗效及预后。

## 病例与方法

1. 病例：入选病例为2009年12月18日至2020年10月29日就诊于中国医学科学院血液病医院的6例IgM型MM患者。所有患者均符合文献[4]的诊断标准，临床分期均按照Durie-Salmon（DS）分期和国际分期系统（ISS）标准。

2. 染色体核型分析及FISH：6例患者均进行G显带核型分析和FISH检查。骨髓标本均采用德国美天旎生物技术有限公司抗CD138磁珠进行浆细胞分选、富集（纯度>90％），应用DNA探针（美国Abbott公司产品）间期FISH（iFISH）技术检测以下改变：1q21扩增、RB1缺失、13q14.3缺失、IgH重排、P53缺失，IgH重排阳性者进行t（4;14）、t（11;14）、t（14;16）、t（14;20）检查。

3. 疗效评价及随访：疗效评价标准参照文献[5]。随访截止时间为2021年1月30日，中位随访时间18.1（3.1～39.6）个月。随访采用查阅患者住院病历和电话随访的形式。5例患者完成随访，1例患者因无法联系失访。

4. 生存指标定义：无进展生存（PFS）期定义为MM诊断至疾病进展、复发或死亡的时间；总生存（OS）期定义为MM诊断至死亡或随访截止时间。

5. 统计学处理：患者实验室检查数据资料以中位数（范围）进行描述。

## 结果

1. 临床特征：本组6例患者中，男4例，女2例，诊断中位年龄为70（59～81）岁。3例轻链为κ型，3例轻链为λ型，DS分期：ⅠA期2例，ⅢA期4例；ISS分期：Ⅱ期4例，Ⅲ期2例。

2. 临床表现及体征：骨痛4例，高黏滞血症3例，淋巴结肿大或肝脾大2例。

3. 常规实验室检查：贫血4例，中位HGB为87（70～131）g/L；白细胞减少1例，中位WBC为5.35（2.59～10.79）×10^9^/L；血小板减少1例，中位PLT为222（30～431）×10^9^/L；肾功能损害2例，中位血清肌酐83.6（53.0～129.6）µmol/L；血清钙水平均正常，中位血清钙2.12（2.11～2.50）mmol/L；中位血清M蛋白39.11（3.61～75.56）g/L；中位血清IgM为69.35（4.35～137.00）g/L，IgG为5.67（2.91～8.89）g/L，IgA为0.46（0.20～1.47）g/L，κ链6.38（2.65～16.22）g/L，λ链2.08（0.04～68.00）g/L。中位β_2_-微球蛋白水平为4.33（2.86～7.75）mg/L。患者具体临床资料见表1。

**表1:** 6例IgM型多发性骨髓瘤患者的临床资料

例号	性别	年龄（岁）	分型	临床分期	溶骨性破坏	高黏滞血症	淋巴结肿大或肝脾大	血M蛋白（g/L）	HGB（g/L）	血清肌酐（µmol/L）	血清钙（mmol/L）	血IgM（g/L）
1	女	67	λ	DS IIIA期，ISS III期	有	无	无	3.61	115	118.3	2.11	4.35
2	女	78	κ	DS IIIA期，ISS II期	有	有	无	75.56	83	75.7	2.50	137.00
3	男	59	λ	DS IIIA期，ISS II期	有	有	无	50.90	72	53.0	2.12	91.10
4	男	81	κ	DS IA期，ISS II期	无	无	无	7.84	131	129.6	2.45	7.28
5	男	62	κ	DS IIIA期，ISS II期	有	有	有	43.17	70	82.0	NA	113.00
6	男	74	λ	DS IA期，ISS III期	无	无	有	35.07	91	85.0	2.11	47.60

注：NA：未检测

4. 细胞形态学：6例患者骨髓细胞形态学均可见浆细胞明显增多，中位浆细胞比例为0.390（0.255～0.590），2例患者可见浆样淋巴细胞增多。4例患者外周血细胞形态学可见浆细胞，中位外周血浆细胞比例0.01（0～0.03）。

5. 常规细胞遗传学检查：染色体核型分析示正常核型5例，低二倍体1例。6例患者均进行FISH检查，2例P53基因缺失阳性，1例1q21扩增阳性（拷贝数为3，阳性率86％），4例RB-1基因缺失阳性。6例患者IgH重排均阳性，其中3例CCND1/IgH融合基因阳性，明确为t（11;14）重排改变。6例患者MYD88 L256P基因突变检测均阴性。

6. 免疫分型：6例患者CD38、CD138、单克隆轻链表达均阳性，为异常浆细胞典型表型。4例患者CD20个别阳性。因例数较少，无法判断有无特殊抗原表达。骨髓检查具体结果见表2。

**表2:** 6例IgM型多发性骨髓瘤患者骨髓检查结果

例号	骨髓浆细胞	染色体核型	FISH检测	免疫表型
1	0.590	低二倍体	P53基因缺失阳性，RB-1基因缺失阳性，IgH基因重排阳性	PAX5（个别+），CD20（个别+），CD3（−），CD5（−），CD138（+），CD38（+），κ（−），λ（+），CD10（−），CyclinD1（−）
2	0.390	二倍体	P53基因缺失阳性，RB-1基因缺失阳性，IgH基因重排阳性	CD138（+），κ（+），λ（−），CD56（−），MUM1（+），CD19（−），CD117（−）
3	0.390	二倍体	IgH基因重排阳性，CCND1/IGH融合基因阳性	CD138（少数+），κ（−），λ（个别+），CD20（个别+），PAX5（个别+），CD3（个别+），CD5（个别+），CD10（−），CD23（−），CyclinD1（个别+）
4	0.430	二倍体	1q21扩增阳性，IGH基因重排阳性	CD138（+），CD38（+），κ（+），λ（−），CD56（−），CD20（部分+），CD3（−）
5	0.290	二倍体	RB-1基因缺失阳性，IgH基因重排阳性，CCND1/IGH融合基因阳性	CD138（+），CD38（+），κ（+），λ（−），CD56（−），CD19（−），CD20（−），CD117（−）
6	0.255	二倍体	RB-1基因缺失阳性，IgH基因重排阳性，CCND1/IGH融合基因阳性	PAX5（个别+），CD20（个别+），CD3（−），CD5（−），CD138（+），CD38（+），κ（−），λ（+），CD10（−），CyclinD1（−）

7. 治疗及生存：所有患者均接受以蛋白酶体抑制剂为主的化疗方案。其中2例采用BCD方案（硼替佐米+环磷酰胺+地塞米松），2例采用IRD方案（伊沙佐米+来那度胺+地塞米松），1例入组临床试验，采用CMP方案（卡非佐米+美法仑+泼尼松），1例采用BD方案（硼替佐米+地塞米松）。6例患者经过诱导治疗后均至少达到部分缓解（PR），2例达到非常好的部分缓解（VGPR），1例达严格意义的完全缓解（sCR），治疗敏感性尚可。随访时间截至2021年1月30日，5例患者可获得完整随访数据，3例后期出现疾病进展而死亡，2例仍维持深度缓解。

## 讨论及文献复习

MM是一种以分泌单克隆免疫球蛋白为特征的恶性浆细胞疾病，常见症状包括相关器官功能损伤，即“CRAB”症状（高钙血症、肾功能不全、贫血、骨病）。而高黏滞血症是MM相对罕见的并发症，但在IgM型MM中较常见，需引起注意。本组有3例（50％）患者初诊时即出现头晕、视物模糊、耳鸣等高黏滞血症表现，可能与患者血清IgM水平较高有关（3例均>90 g/L）。同时，IgM的相对分子质量为950×10^3^，较其他类型免疫球蛋白（如IgG、IgA等）具有更高的轴向长宽比，其独特的分子特征也更易增加血液黏度[6]。国外多个病例报道提示，早期出现高黏滞血症的IgM型MM患者较高危，建议尽早进行血浆置换，并积极全身治疗，减少异常单克隆免疫球蛋白的产生，尽快达到疾病深度缓解[7]–[9]。

华氏巨球蛋白血症（Waldenstroms macroglobulinemia，WM）是一种淋巴浆细胞肿瘤，以分泌IgM型单克隆免疫球蛋白为特征，常表现为骨髓浸润、高黏滞血症、淋巴结肿大或肝脾大等，临床表现与IgM型MM有许多相似之处，难以鉴别。但溶骨性病变是MM特征性表现，在WM患者中罕见，有助于两者的区分。其次，WM患者大多有MYD88 L256P基因突变，而MM中常无这一突变[10]。此外，IgM型MM患者IgH易位阳性率极高，其中t（11;14）最为常见，可在40％患者中发现这一遗传学改变[6],[11]。而WM患者最常见的细胞遗传学改变为6q−，几乎不伴IgH易位，因此检测IgH易位与6q−对鉴别WM与IgM型MM也有一定意义。

国外有研究回顾性分析17例IgM型MM患者，可在4例患者外周血中发现大量浆细胞，并有2例进展为浆细胞白血病，提示IgM型MM患者较常见亚型进展为浆细胞白血病的风险高[12]。本组患者中，有4例可在血涂片中发现浆细胞，虽然外周血浆细胞比例较低（均<5％），但不能否认IgM型MM髓外进展风险高，提示我们对IgM型MM患者应进行细致的血涂片检查或外周血流式细胞术检测，避免浆细胞白血病的漏诊。此外，4例外周血浆细胞阳性的患者多伴有t（11;14），与国外报道结果一致，提示t（11;14）可能是MM进展为浆细胞白血病的高危因素[12]。

在治疗方面，国外一项大型多中心研究纳入134例IgM型MM患者，发现这一亚型对传统化疗药物、蛋白酶体抑制剂等均较为敏感，且预后尚可，中位OS时间可达61个月，与常见MM亚型无明显区别[6]。既往国内有研究报道4例IgM型MM，提示这一罕见亚型进展迅速，预后相对较差，可能与新药应用较少有关[13]。本组6例患者均接受以蛋白酶体抑制剂等新药为基础的治疗，治疗敏感性较好，疗效均至少获得PR，与国外研究结果类似[6],[12]。但本组患者病例数较少，且部分患者随访时间较短，无法准确评估IgM型MM患者的生存期。自体移植是MM系统治疗中的重要一环，但有研究表明对IgM型MM患者进行自体移植并不能改善预后，生存期相对较短[14]。此外，有研究提示Bcl-2抑制剂对携带t（11;14）重排的MM患者具有较好疗效[15]，因此维奈托克治疗IgM型MM可能会改善其预后，并延长患者生存期。

IgM型MM非常罕见，具有一定的临床特点，除具有骨髓瘤典型临床表现外，也可伴高黏滞血症、淋巴结肿大或肝脾大等淋巴浆细胞肿瘤的特征表现。同时，t（11;14）易位是IgM型MM最常见的细胞遗传学异常。此外，在IgM型患者外周血中常能发现浆细胞，提示这一亚型髓外进展风险高。在治疗反应上，IgM型MM对目前常用治疗药物均较为敏感，疗效尚可。综合国外既往报道，与常见亚型相比，IgM型MM未显示明显预后不良。
